# *KIR/HLA* Gene Profile Implication in Systemic Sclerosis Patients from Mexico

**DOI:** 10.1155/2019/6808061

**Published:** 2019-01-06

**Authors:** Andrea Carolina Machado-Sulbaran, María Guadalupe Ramírez-Dueñas, José Eduardo Navarro-Zarza, José Francisco Muñoz-Valle, Francisco Mendoza-Carrera, Christian Johana Baños-Hernández, Isela Parra-Rojas, Margarita Montoya-Buelna, Pedro Ernesto Sánchez-Hernández

**Affiliations:** ^1^Universidad de Guadalajara, Laboratorio de Inmunología, Departamento de Fisiología, Centro Universitario de Ciencias de la Salud, 44340 Guadalajara, Jalisco, Mexico; ^2^Hospital General Dr. Raymundo Abarca Alarcón, Departamento de Medicina Interna-Reumatología, 39019 Chilpancingo, Guerrero, Mexico; ^3^Universidad de Guadalajara, Instituto de Investigación en Ciencias Biomédicas, Departamento de Biología Molecular y Genómica, 44340 Guadalajara, Jalisco, Mexico; ^4^Instituto Mexicano del Seguro Social, División de Medicina Molecular, Centro de Investigación Biomédica de Occidente, 44340 Guadalajara, Jalisco, Mexico; ^5^Universidad Autónoma de Guerrero, Laboratorio de Investigación de Obesidad y Diabetes, Facultad de Ciencias Químico Biológicas, 39090 Chilpancingo, Guerrero, Mexico

## Abstract

**Introduction:**

Systemic Sclerosis (SSc) is an autoimmune, inflammatory, and multisystemic disease characterized by the presence of autoantibodies and fibrosis. The pathogenesis involves the interaction between immune system cells such as macrophages, NK cells, T cells, and B cells. Killer-cell Immunoglobulin-like Receptors (KIR) are expressed in NK cells and some T cell subsets that recognize HLA class I molecules as ligands and are involved in regulating the activation and inhibition of these cells. The *KIR* family consists of 14 genes and two pseudogenes; according to the gene content, the genotype could be AA and Bx. The aim of this study was to evaluate the association between *KIR/HLA* genes and genotypes with SSc and the clinical characteristics.

**Methods:**

We included 50 SSc patients and 90 Control Subjects (CS). Genotyping of *KIR*, *HLA-C*, *-Bw4*, and *-A*^∗^*03/*^∗^*11* was made by SSP-PCR.

**Results:**

In SSc patients, a higher frequency of *KIR2DL2* (*p* = 0.0007, *p*′ = 0.011), *KIR2DS4del* (*p* = 0.001, *p*′ = 0.021), and *HLA-C2* (*p* = 0.02, *p*′ = 0.09) was found. This is the first study to evaluate the frequency of *HLA-A*^∗^*03/*^∗^*11* in SSc patients, of which a low frequency was found in both groups. Compound genotypes *KIR2DL2+/HLA-C1+* or *KIR2DL2+/HLA-C2+* have a higher frequency in SSc patients. The Bx genotype was the most frequent and was associated with risk to SSc (*p* = 0.007, OR = 3.1, 95% CI = 1.4–7.9, *p*′ = 0.014). The genotypes with a higher *iKIR* number than *aKIR* (*iKIR* > *aKIR*) were found in all individuals; genotypes with 7-8 *iKIR* genes were increased in SSc patients. We do not find an association between the *KIR* genes with the clinical characteristics.

**Conclusion:**

The results suggest that *KIR2DL2* and *2DS4del* could have a risk role in the development of SSc, but not with clinical manifestations.

## 1. Introduction

Systemic Sclerosis (SSc) is a chronic and autoimmune disease, characterized by vascular dysfunction and damage, alteration in angiogenesis, inflammation, cytokine release, and fibrosis. The damage in the endothelial cells can be mediated by autoantibodies, viruses, oxidative stress, or by granzymes and perforins. It mainly affects the skin, gastrointestinal tract, lungs, and kidneys. According to the extent of skin fibrosis, SSc can be classified as limited (lcSSc) or diffuse (dcSSc) [1]. The triggering agents are not entirely established; however, environmental (silica, asbestos, organic solvents, and industrial emissions), infectious (mainly Cytomegalovirus and Epstein Barr virus infections), and genetic factors (mainly *HLA* class I and II alleles) are commonly involved in the susceptibility and disease development [2, 3].

Natural Killer (NK) cells play an important role in the innate and adaptive immune response by the secretion of cytokines and chemokines or by cytotoxicity. NK cells, in addition to T cells, have been implicated in different autoimmune processes [4]. The functions of NK cells and some subpopulations of T cells are regulated by membrane receptors such as the Killer-cell Immunoglobulin-like Receptors (KIR), which recognize as ligands HLA (Human Leukocyte Antigen) class I molecules (-A, -B, -C, -F, -G). The KIR extracellular region can have two (KIR2D) or three (KIR3D) domains, and the intracytoplasmic tail can be long tailed (L; e.g., KIR2DL and KIR3DL) with an inhibitory function or short tailed (S; e.g., KIR2DS and KIR3DS) with an activating function. The KIR/HLA-I interaction occurs between the extracellular domains and the peptide residues of the HLA-I molecule. Its affinity is influenced by the amino acid positions [5]. The HLA-C KIR ligands are classified into the -C1 (position 77 has a serine and position 80 an asparagine) ligand of KIR2DL2, 2DL3, 2DS2, and 2DS4 and the -C2 (position 77 has an asparagine and position 80 a lysine) ligand of KIR2DL1, 2DL2, 2DL3, 2DS1, 2DS4, and 2DS5. *HLA-B* alleles are classified into *-Bw4* and *-Bw6* based on the amino acid positions 77-83. The products of *KIR3DL1* gene variants are associated with HLA alleles with *-Bw4* motifs (*HLA-A^Bw4^*, *-Bw4^Iso80^*, *-Bw4^Thr80^*). With regard to HLA-A, it has mainly been described that HLA-A^∗^03 and -A^∗^11 are ligands of KIR3DL2 and, in addition, HLA-A^∗^11 is a ligand of KIR2DS4 [5, 6].

*KIR* genes are located in the leukocyte cluster receptor, at chromosome 19q.13.4. This family includes genes encoding seven inhibitory receptors (*KIR2DL1*, *2DL2*, *2DL3*, *3DL1*, *3DL2*, *3DL3*, and *2DL5*), six activating receptors (*2DS1*, *2DS2*, *2DS3*, *2DS4*, *2DS5*, and *3DS1*), one receptor with both functions (*2DL4*), and two pseudogenes (*2DP1* and *3DP1*). *KIR3DL2*, *3DL3*, *2DL4*, and *3DP1* are considered framework genes and usually are present in all individuals [5, 7]. The *KIR* haplotype is defined, depending on the gene content, as A or B. The B haplotype is characterized by the presence of at least one of its exclusive genes, *KIR2DL5*, *2DS1*, *2DS2*, *2DS3*, *2DS5*, or *3DS1*. The A haplotype is identified by the absence of all exclusive *KIR* genes from the B haplotype. In addition, this characteristic is used to define the AA genotype in subjects. Because we cannot distinguish between AB and BB genotypes, any of these are called Bx [8].

Studies in different populations have reported associations of *KIR* genes and *KIR/HLA-I* genotypes with SSc susceptibility: *KIR2DS2+/2DL2-* in Germany [9]; *KIR2DS1+/2DS2-* and *2DS1+/HLA-C2+* in Canada [10]; *2DS3+*, *2DS2+HLA/-C1+*, and *2DL2+/HLA-C1+* in Turkey [11]; and *3DL1+/HLA-Bw4^Thr^-* in Iran [12]. On the other hand, other studies have shown the association of *KIR* genes and *KIR/HLA-I* genotypes with lower risk to SSc: *2DL2+* in Brazil [13] and *2DL3+* in Turkey [11]. In the Mexican population, there are only a few studies performed in SSc patients and the role of *KIR* in SSc has not been evaluated. Therefore, the aim of our study was to evaluate the association between *KIR* and *HLA-I* genes with SSc and the clinical manifestations in patients from Guerrero state (southern Mexico).

## 2. Material and Methods

### 2.1. Studied Populations

We included 50 patients with SSc and 90 Control Subjects (CS) without familiar autoimmune antecedents. A rheumatologist evaluated all patients and classified for SSc according to the 2013 American College of Rheumatology and The European League Against Rheumatism (ACR/EULAR) criteria. The patients were recruited by continuing inclusion during January 2016 to June 2017, in the Hospital General de Chilpancingo Dr. Raymundo Abarca Alarcón, Guerrero, México. The patients were matched with CS in a 1 : 2 ratio, considering age and gender. All subjects were from Guerrero state (southern Mexico), at least until the third generation. Due to the genetic heterogeneity of the Mexican population previously demonstrated in *KIR* and *HLA* genes [14, 15], we did not include patients from other regions to conserve the ethnic homogeneity and to avoid, as much as possible, this influence in our results. The study was part of a protocol about genetic associations in Mexicans, and informed consent was obtained from each individual. This project was conducted according to the Helsinki Declaration and approved by the Committee of Biosecurity and Ethics of the Centro Universitario de Ciencias de la Salud at the Universidad de Guadalajara and at the Universidad Autónoma de Guerrero, México.

### 2.2. KIR and HLA Genotyping

Genomic DNA was obtained from peripheral blood, using EDTA as an anticoagulant. For the DNA extraction, we utilized the modified technique of Miller et al. [16]. The DNA samples were quantified in a NanoDrop (Thermo Fisher Scientific, USA) at 260/280 nm wavelength, and work dilutions were settled at 100 ng/*μ*L.

The identification of *KIR* genes (*2DL1*, *2DL2*, *2DL3*, *3DL1*, *3DL2*, *2DS1*, *2DS2*, *2DS3*, *2DS4*, *2DS5*, *3DS1*, *2DP1*, *3DL3*, *2DL4*, *2DL5*, and *3DP1*) was made by Sequence Specific Primer Polymerase Chain Reaction (SSP-PCR). The primers and conditions were established according to the method described by Vilches et al. [17]. The unique *KIR* genotype profile was analyzed and confirmed in duplicate. For *KIR2DS4*, we identify the *full* gene and *del* variant, which was considered for the group assignment. *HLA-C* (*-C1* and *-C2*) typing was made by SSP-PCR using the methodology established by Hiby et al., [18]. *HLA-Bw4* (*-A^Bw4^*, *-Bw4^Iso80^*, and *-Bw4^Thr80^*) were identified by SSP-PCR using primers described by Tajik et al. [19] and the conditions described by Omar et al. [20]. We used 100 ng of DNA in each reaction. The electrophoresis of *KIR* and *HLA-C* PCR products were done in 3.0% agarose gel (Vivantis®) and the *HLA-Bw4* PCR products in 2.0% agarose gel (Vivantis®). All gels were prepared with Ultrapure 0.5x TBE buffer (Life Technologies®) and stained with SYBR Green Safe (Invitrogen®). A Kodak® Gel Logic 112 imaging system was used for visualization and photography. Genotyping of *HLA-A*^∗^*03* and *-A*^∗^*11* was made by SSP-PCR, according to Kurz et. al., [21]. With this strategy, alleles of both allelic groups are coamplified from two PCR mixes and the products visualized in a 6% polyacrylamide gel stained with silver nitrate. Subsequently, to discriminate between *HLA-A*^∗^*03* and *-A*^∗^*11*, samples positive for *HLA-A*^∗^*03/-A*^∗^*11* were purified and genotyped by sequence-based typing (SBT) of a fragment containing exons 2 and 3 of the *HLA-A* gene. Sequencing was performed by using the Dye Terminator Cycle Sequencing (DTCS) Quick Start Kit (Beckman Coulter, Fullerton, CA) and, after dye terminator washing, samples were sequenced in a Genetic Analyzer Beckman Coulter CEQ8800 instrument according to the manufacturer's recommendations. For allele assignment, all sequences were analyzed in the Chromas Software Version 2.4.6 and online blasted with those reported in the IMGT-HLA database.

### 2.3. Statistical Analysis

The carrier frequency (CF) estimation of each *KIR* and *HLA* gene was done using direct counting. The *KIR* and *HLA* gene frequency (GF) was determined by Bernstein's formula $GF=1-\sqrt{\left(1-F\right)}$ (using CF) [22]. The differences in clinical parameters, *KIR/HLA* genes, and genotype frequencies were analyzed by Fisher's exact test and adjusted *p*′ with Holm-Bonferroni correction for multiple comparisons. Hardy-Weinberg equilibrium was evaluated in both study groups according to the *KIR* genotype profile. Comparisons between *KIR* and *HLA* genes with the clinical characteristics and clinical evaluation scales were done with multiple comparisons, and each group was analyzed by Fisher's exact test. GraphPad Prism Software Version 6 was used for all analyses and the statistical power was calculated using the program Power and Sample Size Calculation version 3.1.6 [23]. The results were considered significant when *p* < 0.05, and the statistical power was between 82 and 100%. The linkage disequilibrium (LD) in *KIR* genes was analyzed using Cramer's *V* statistic [22], which was calculated from the contingency table of presence/absence, and we refer to this statistic as $Wn^{*}=\left(ad-bc\right)/\sqrt{\left(\left(a+b\right)\left(c+d\right)\left(a+c\right)\left(b+d\right)\right)}$. *Wn*^∗^ value was calculated only in the *KIR* genes associated with the disease. Chi square was used to test the association in pairs of genes, and *p* < 0.05 was considered statistically significant.

## 3. Results

### 3.1. Demographic and Clinical Features

A total of 50 SSc patients were included, 44 female (88%) and six male (12%). 14% of the patients have dcSSc (all women), whereas 86% of the patients have lcSSc. SSc patients have a median age of 48.7 years at the inclusion time (Table 1). Regarding the risk factor exposure, the majority of the SSc patients were exposed to wood smoke (78%; *p* < 0.0001, OR = 6.8, 95% CI = 3.0–14.9) mainly from cooking; they were also exposed to fertilizers (38%), organic solvents (12%), silica (8%), and asbestos (2%). The more frequent clinical manifestations are shown in Table 1. Concerning the clinical evaluation, at the inclusion time, the median of HAQ (Health Assessment Questionnaire disability index) was 0.4 and median of MRSS51 (Modified Rodnan Skin Score) was 7.3 (Table 1).

### 3.2. *KIR* and *HLA* Genes

The distribution of *KIR* and *HLA* class I genes in SSc patients and CS is shown in Table 2. The *KIR2DL2* gene frequency was higher in SSc patients compared with CS (*p* = 0.0007, OR = 3.6, 95% CI = 1.7–7.3, *p*′ = 0.011). According to the methodology used, we were able to distinguish the *KIR2DS4* gene variants: *2DS4full* and *2DS4del*. Taking into consideration whether individuals have or do not have the *full* or *del* gene variant, four groups were defined: (a) the individuals with both variants (*full/del*), (b) those with only the *full* gene (*full*), (c) those with only the *del* variant (*del*), and (d) those who are negative for *KIR2DS4* (*neg*). The *KIR2DS4full* frequency was lower in SSc patients than in CS (*p* = 0.02, OR = 0.3, 95% CI = 0.1–0.7, *p*′ = 0.09), whereas the *2DS4del* frequency was higher in SSc patients than in CS (*p* = 0.001, OR = 7.4, 95% CI = 1.6–35.2, *p*′ = 0.021). Individuals with *full/del* and the *KIR2DS4neg* have similar frequencies in both groups (Table 2). Concerning *HLA* class I gene distribution, *HLA-A^Bw4^* and *HLA-C2* were more frequent in SSc patients than in CS (Table 2) without significant differences. Regarding *HLA-A*, we found 7 SSc patients (14%) and 12 CS (13.3%) positive to *-A*^∗^*03/*^∗^*11*. In SSc patients, 8% are positive to *-A*^∗^*03* and 6% to *-A*^∗^*11*. In CS, 11.1% are positive to *-A*^∗^*03* and 1.1% to *-A*^∗^*11* without significant difference (Table 2).

### 3.3. Combinations of *KIR* Genes and *KIR*/*HLA* Genotypes

The *KIR* gene combinations involve the presence/absence of the genes previously associated with SSc in this study and in previous studies (*2DL2*, *2DS4*, *2DS2*, and *2DS1*). Analysis shows an increased frequency of *2DL2+/2DS4del+* in SSc patients compared with CS. In SSc patients, a low frequency of *2DL2-/2DS4del-* (*p* = 0.0005, OR = 0.2, 95% CI = 0.1–0.6, *p*′ = 0.005), *2DL2-/2DS4full+/2DS4del-* (*p* = 0.0003, OR = 0.2, 95% CI = 0.08–0.5, *p*′ = 0.003), *2DL2+/2DS2-* (*p* < 0.0001, OR = 17.1, 95% CI = 3.7–79.2, *p*′ = 0.0009), and *2DL2-/2DS2-* (*p* = 0.0007, OR = 0.3, 95% CI = 0.1–0.6, *p*′ = 0.006) was found. The other *KIR* gene combinations do not show significant differences between both groups. Due to the differences observed in the frequencies of *KIR2DL2* and *KIR2DS2* in the SSc patients (Table 2) and the preferential association between these genes reported in other investigations [24], we calculated the LD of these genes. In CS, a strong LD is observed (Wn^∗^ = 0.9540, *p* < 0.0001), while in SSc patients a decreased LD is observed (Wn^∗^ = 0.5625, *p* < 0.0001).

Taking into consideration the presence of each of the *KIR* genes with their specific *HLA-I* ligand gene, the compound *KIR/HLA* genotypes were evaluated. The comparison of the compound *KIR/HLA* genotypes of SSc patients and CS with a statistically significant difference is shown in Table 3. In SSc patients, the frequency of the compound genotypes *2DL2+/HLA-C2+* was increased (*p* = 0.01, OR = 2.8, 95% CI = 1.3–5.8, *p*′ = 0.045). On the other hand, the frequency of *2DL2-/C1+* (*p* = 0.001, OR = 0.3, 95% CI = 0.1–0.6, *p*′ = 0.004), *2DL2-/HLA-C2-* (*p* < 0.0001, OR = 0.1, 95% CI = 0.03–0.4, *p*′ = 0.0004), *2DS4full+/HLA-C1+* (*p* = 0.003, OR = 0.3, 95% CI = 0.2–0.7, *p*′ = 0.01), and *2DS4del-/HLA-C2-* (*p* = 0.0005, OR = 0.1, 95% CI = 0.04–0.5, *p*′ = 0.002) was lower in SSc patients (Table 3). The other *KIR/HLA* gene combinations that involve the presence of *HLA-A^Bw4^*, *-Bw4^Iso^*, *-Bw4^Thr^*, and -*A*^∗^*03/*^∗^*11* and their respective *KIR* did not show a significant difference between both groups.

### 3.4. *KIR* Genotypes

In this study, 35 genotypes were found in all individuals, including 24 genotypes in SSc and 24 genotypes in CS. Eleven genotypes were exclusive to the SSc patients, 11 were exclusive to the CS, and 13 were shared between both groups (Table 4). A novel genotype (not reported in the Allele Frequency Database [8]) was found in an SSc patient. The AA genotype was less frequent in SSc patients (16%) than in CS (38.9%), whereas the Bx genotype was more frequent in SSc patients (84%) than in CS (61.1%) (*p* = 0.007, OR = 3.1, 95% CI = 1.4–7.9, *p*′ = 0.014). In particular, the frequency of the genotype with ID 1 (as reported in the Allele Frequency Database [8]) was lower in SSc patients (14%) than in CS (37.8%) (*p* = 0.03, OR = 0.3, 95% CI = 0.1–0.7, *p*′ = 0.02). The genotype with ID 19 (as reported in the Allele Frequency Database [8]) was found only in patients (14%) (Table 4).

**Figure 1:**
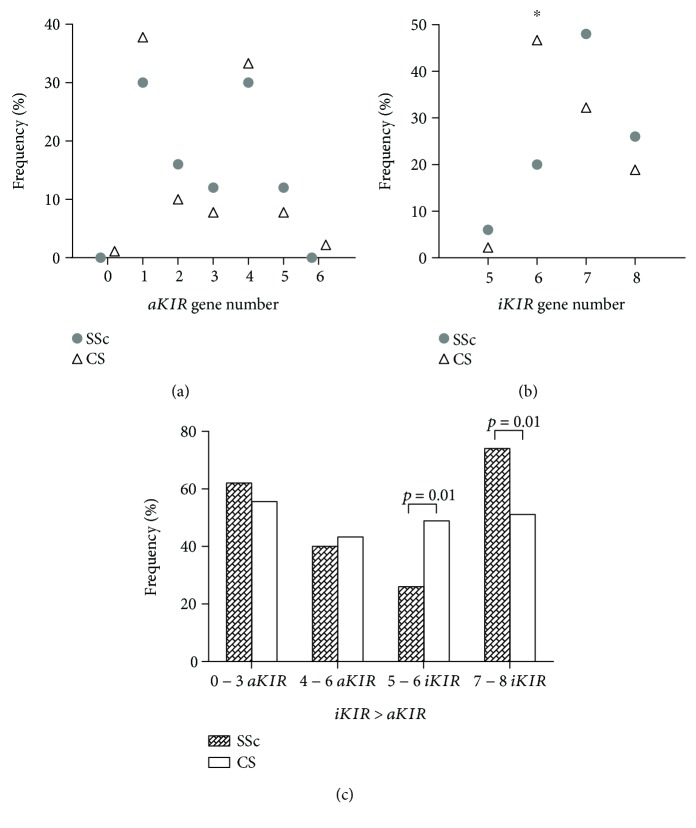
Distribution of activating/inhibitory *KIR* gene number between SSc patients and CS. (a) The number of activating *KIR* (*aKIR*) gene number percentage in SSc patients (gray circle) and CS (white triangles) is shown. (b) The number of inhibitory *KIR* (*iKIR*) gene number percentage in SSc patients (gray circle) and CS (white triangles) is shown. (c) The genotypes with higher *iKIR* than *aKIR* (*iKIR* > *aKIR*) is indicated according to the number of *iKIR* and *aKIR* genes present. SSc: Systemic Sclerosis; CS: Control Subjects; ^∗^*p* = 0.002 based on Fisher's exact test and *p*′ = 0.008, based on Holm-Bonferroni correction for multiple comparisons.

According to the genotype, the number of activating/inhibitory *KIR* genes was evaluated (Figure 1). The percentage of genotypes with 2, 3, and 5 activating *KIR* genes (*aKIR*) were higher in SSc patients than in CS, whereas genotypes with 1 and 4 *aKIR* genes have a lower percentage in SSc patients (Figure 1(a)), without showing statistical significance. In addition, the genotypes with 7 and 8 *iKIR* genes (*iKIR*) were more frequent in SSc patients (Figure 1(b)); however, it was not statistically significant. The percentage of individuals with 6 *iKIR* genes was lower in SSc patients than in CS (*p* = 0.002, OR = 0.3, 95% CI = 0.1–0.6, *p*′ = 0.008).

All the genotypes found in our studied groups have higher *iKIR* genes than *aKIR* genes (*iKIR* > *aKIR*). We established two groups according to the *aKIR* gene number: 0-3 *aKIR* and 4-6 *aKIR*. The frequency of individuals with 0-3 *aKIR* and 3-6 *aKIR* does not show a significant difference between SSc patients and CS (Figure 1(c)). We established two groups according to the *iKIR* gene number: 5-6 *iKIR* and 7-8 *iKIR*. The frequency of individuals with 7-8 *iKIR* genes was higher in SSc patients than in CS (*p* = 0.01; OR = 2.7; 95% CI = 1.3–5.8, *p*′ = 0.02), and the percentage of individuals with 5-6 *iKIR* genes was lower in SSc patients than in CS (*p* = 0.01; OR = 0.3; 95% CI = 0.2–0.8, *p*′ = 0.02).

### 3.5. *KIR* and *HLA* Genetic Associations with the Presence of Clinical Manifestations

The *KIR* genes associated with the disease (*KIR2DL2* and *2DS4del*) were analyzed with the following clinical manifestations: sclerodactyly, musculoskeletal damage, Raynaud's phenomenon, inflamed fingers, telangiectasia, digital ulcers, calcinosis, esophageal dysfunction, interstitial lung disease, and pulmonary arterial hypertension. In almost all clinical manifestations, we found a higher frequency of *2DL2* than the other genes (Table 5); however, there was no significant difference, probably due to the number of patients. In addition, our evaluation shows that the presence of *HLA*, *KIR/HLA* genotypes, and *iKIR/aKIR* number does not have an association with the clinical manifestations, clinical evaluation (HAQ levels and MRSS51 score), and exposure to environmental risk factors (silica, organic solvents, fertilizers or, wood smoke).

## 4. Discussion

Systemic Sclerosis (SSc) is an autoimmune disease more frequent in women than in men, according to the EUSTAR (EULAR Scleroderma Trials and Research group) records which showed a 5-10 : 1 ratio depending on the population. A female predominance was found in our studied patients (7.3 : 1), which is within the range of the EUSTAR records. In patients from Mexico City, there was also a higher prevalence of women with SSc; however, there was a dissimilar proportion compared to that of our study (9 : 1) and the majority of our patients have lcSSc, as previously observed in patients from Mexico City [2, 25]. Concerning the environmental factors, silica exposure was possibly involved with construction-related occupations. The organic solvent was suspected in patients with occupational exposure to cleaning products and trichloroethylene derivatives or in those working in the paint industry [26].

In this study, most of the patients live in rural areas and exposure to wood smoke is a significant risk factor. The exposed subjects are 6.8 times more likely to develop the disease compared to those who are not exposed. It is currently estimated that people exposed to wood smoke are mainly from the rural areas. Until now, welding smoke and industrial emission particles have been associated with risk to SSc development; however, the role of wood smoke in SSc susceptibility has not been described [2, 3]. Previous studies have shown that wood smoke exposure can lead to NF-*κ*B overactivation; increased serum levels of TNF-*α*, IFN-*γ*, IL-1*β*, IL-18, IL-6, IL-21, and CCL2; and higher numbers of neutrophils, monocytes, and lymphocytes; as well as lower expression of MMP-9 and MMP-12. This mechanism induces chronic systemic inflammation and fibrosis. In addition, wood smoke produces oxidative damage, which could affect proteins and lipids (lipid peroxidation) or induce DNA damage, favoring autoantibody production [26–31]. In this study, we did not evaluate the autoantibody positivity; however, future studies could evaluate the relationship between wood smoke and the presence of autoantibodies in SSc patients.

In SSc immunopathology, various immune cells are involved; with regard to the NK cell, there are discrepant reports concerning the number and functions, which are probably related to the disease stage (inflammatory, fibrotic, or atrophic) and different clinical complications (CREST syndrome, skin fibrosis extension, vascular involvement, and lung damage). The decrease of NK cell numbers in peripheral blood is attributed to the infiltration in affected tissues; nevertheless, others studies report an increased NK cell number in dsSSc. In any of the cases, NK cells from SSc patients exhibited phenotypes characterized by altered cytokine production, diminished natural cytotoxicity, and decreased granzyme B release [32, 33]. The NK cell functions are controlled by inhibitory and activating receptor-ligand interactions. The signals from inhibitory receptors and HLA ligands are considered a mechanism for NK cell self-tolerance in a process named NK cell education, which is essential for tolerance to self-components. Strikingly, the NK cell licensing is reversible and the responsiveness of mature NK cells may adapt to environmental changes; for this reason, diverse studies have analyzed *KIR* genes in various autoimmune pathologies, such as psoriasis vulgaris and rheumatoid arthritis (RA) [7, 34].

This is the first study to evaluate the association of *KIR*/*HLA* genes and genotypes with SSc in the Mexican population. In this study, the framework genes (*KIR3DL2*, *3DL3*, *2DL4*, and *3DP1*) and pseudogenes (*2DP1* and *3DP1*) were found in almost all individuals. *KIR* gene frequencies in CS were similar to a previous report from the Guerrero population [14]. In the patients studied, *KIR2DL2* presence was associated with risk to SSc; in contrast, this gene has been reported with a lower risk of SSc development in Brazilian patients. However, in western Mexico, *2DL2* has also been associated with the risk for RA and response to treatment; this could suggest that in the Mexican population, *2DL2* is a gene associated with susceptibility to autoimmunity [13, 35].

Different studies have postulated that the increased expression of KIR2DL2 induces lower NK cell activation (mainly in viral infection), and the blockade of this receptor can favor cytotoxicity and cytokine secretion. KIR2DL2 could inhibit the signals from activating KIR or other activating receptors [36]. Thus, the NK cells of individuals with KIR2DL2 could have affected the antifibrotic function of the NK cells, characterized by the secretion of cytotoxic substances that induce cell lysis and the release of cytokines such as IFN-*γ*. It has been reported that IFN-*γ* production by NK cells inhibits liver fibrosis; therefore, altered NK responses could contribute to fibrogenesis [4, 32, 34]. In autoimmune diseases and particularly in SSc, the role of *KIR2DL2* gene products is not fully established; however, it is possible that it is overexpressed and has high affinity with their ligands (HLA-C1 and -C2). This could be influential in the loss of the NK cells' antifibrotic effect and eventually contribute to fibrosis progression in SSc.

In addition, a strong LD between *KIR2DS2* and *KIR2DL2* in CS was found, while in SSc patients the LD was decreased. A possibility is that the patients have a *KIR2DS2* allele that is not reported and that is not amplified with the primers used (must be confirmed by sequencing techniques). Moreover, NK cell receptors can only diversify through meiotic mutation and recombination processes; therefore, an incident during these processes affects the lineage distinctions and even disturbs the segregation of these receptors, as is described in other *KIR* gene combinations [37].

The KIR2DS4 protein can be encoded by 33 alleles, of which some code for a complete protein (2DS4full) and others for a truncated protein (2DS4del), for having the loss of 22 pb (mutations in exon 5 or 7). Human *2DS4del* was initially called KIR1D, and its function is still unknown. However, the changes in the protein structure can affect their activating function [38]. In SSc patients, the *2DS4del* presence was associated with risk to SSc, while *2DS4full* was associated with reduced risk. In autoimmune diseases, *2DS4full* was associated with a lower chance of response to methotrexate in RA patients [39]. In other medical conditions like a kidney transplant, *KIR2DS4* gene variants are associated with the risk of rejection to kidney transplantation [40]. *KIR2DS4full* was associated with high survival after one year in patients with hematopoietic cell transplantation. Furthermore, in this group of patients *2DS4del* was associated with risk to graft-versus-host disease [41] and also with susceptibility to syphilis in China [42]. It has been believed that KIR2DS4del is a nonfunctional protein because it is not anchored on the surface of the cell membrane. Nevertheless, it can be secreted in a soluble form and bind to its ligands (HLA-A^∗^11 and -C1/-C2) avoiding the recognition for other receptors or acting as a ligand to other receptors, which can trigger the activation of other types of cells that participate in the disease immunopathology [43].

In *HLA* class I genes, *HLA-A^Bw4^* has been previously associated with risk to SSc in Iranian subjects [12]. In a previous study in Mexican patients, the genes *-C*^∗^*12:03/-B18:01* and *-B*^∗^*08:01* were reported as a genetic risk to SSc, whereas *-C*^∗^*07:02*, *-C*^∗^*07:02+/-B*^∗^*39:05*, and *-B*^∗^*39:06* were reported with reduced risk to dcSSc development [25]. In Brazilian patients, alleles of *HLA-I* have been associated not only with SSc development (*-A*^∗^*30*, *-A*^∗^*32*, *-B*^∗^*57* and *-Cw14*) but also with medical complications, such as pulmonary fibrosis (*-Cw*^∗^*0602*) and pulmonary arterial hypertension (*-C*^∗^*04*) [44]. Thus, the presence of *HLA* class I genes may have a role in SSc susceptibility and development. None of the *HLA* evaluated in this research are associated with the SSc; therefore, future studies should evaluate other *HLA* groups in the Mexican population.

The combined genotypes associated with risk (*2DL2+/2DS2-*) or decreased risk of SSc (*2DL2-/2DS4del-*, *2DL2-/2DS4full+/2DS4del*-, and *2DL2-/2DS2-*), have not been reported in any other investigation. Combined genotypes associated with risk to SSc are *2DS2+/2DL2*- in Germany and Brazil and *2DS1+/2DS2-* in Canada [9, 13]. The analysis of *KIR/HLA* compound genotypes demonstrates that *KIR2DL2+/HLA-C1+* and *2DL2+/-C2* are associated with risk in SSc patients of our population. The compound genotype *2DL2+/HLA-C1+* had already been associated with SSc in patients from Turkey and *2DS1+/HLA-C2+* in Germany [9, 11]. The other combinations found have not been associated with autoimmune pathologies. And *2DS4del+/HLA-C1+* and *-C2+* have been associated with high transmission of HIV from Black South African mothers to children during pregnancy.

Regarding *KIR* genotypes, the Bx genotype was more frequent in SSc patients; in particular, the Bx genotype with ID 19 (according to the Allele Frequency Database [8]) was found only in SSc patients, whereas the AA genotype with ID 1 (according to the Allele Frequency Database [8]) was associated with lower risk to SSc development. Several investigations have confirmed that the AA genotype (predominantly inhibitor) has a certain protective effect to autoimmune diseases, such as RA, while the Bx genotype (predominantly activator) represents a risk to autoimmunity. Also, the Bx genotype with ID 19 was associated with risk for SSc in patients from Iran and risk for RA in patients from western Mexico[7, 12, 36]; however, we do not get a significant difference, probably due to the number of patients. A particularity of the genotype with ID 19 is that it is only distinguished from the genotype AA with ID 1 by the presence of *KIR2DL2*. It is important to consider that in addition to the *KIR2DL2* association with the SSc, we confirm that the development of some clinical characteristics, such as sclerodactyly, musculoskeletal damage, inflamed fingers, and digital ulcers are more frequent in patients with *2DL2*, in which the presence or absence of *2DS4* or any of its genetic variants was not involved. The involvement of *KIR2DL2* in the development of various clinical characteristics is not defined; however, if the antifibrotic activity of the NK cells is inhibited, many other mechanisms could stimulate fibrosis and disease progression in conjunction with the various clinical complications. It is important to consider that we could not obtain a significant OR due to the small number of patients; future studies could evaluate the involvement of *KIR2DL2* with the clinical characteristics of the disease in a larger group of SSc patients.

The influence of the activating/inhibitory *KIR* gene number in SSc susceptibility was demonstrated. The genotype with 7-8 *iKIR* genes was increased in SSc patients. The role of *iKIR* and *aKIR* gene number in autoimmune diseases is not well established; however, the effect of *KIR* gene number variation on NK cell education and the ability to respond to infections (mainly viral infections) is widely known. NK cell education via KIR is a process dependent on the interaction with HLA-I molecules, and for this reason, they must be analyzed altogether to estimate their potential involvement [45]. It is not clear how NK education can affect SSc immunopathology. However, the NK cell action depends on the balance between the signals from inhibitory and activating KIR receptors for different ligand specificities and the difference in the *iKIR* and *aKIR* numbers could affect its cytotoxic activity or cytokine secretion capacity.

In summary, this study provides a comprehensive assessment of the *KIR* and *HLA* genetic susceptibility with SSc in a southern Mexican mestizo population. The differences found in the *KIR* and *KIR/HLA* frequencies in our SSc patients in comparison to the SSc patients from other populations can be produced by genetic admixture and adaptation processes influenced by infectious agents and the environmental factors of the different regions and continents [7].

One limitation of this study is the low number of patients that we included; however, SSc has a low incidence rate. In addition, we consider preserving the genetic homogeneity more valuable than including patients from other regions because we previously demonstrated the genetic diversity of *KIR* in various Mexican regions [14]. Future studies should be done in the regions of other countries. Moreover, alternative techniques should be used to determine the specific alleles associated with the disease, evaluate *KIR* implication in the clinical manifestation in a larger group of SSc patients, and evaluate the expression of KIR in infiltrating cells in biopsies from SSc patients.

## Figures and Tables

**Table 1:** Demographic and clinical characteristics of SSc patients and CS.

	SSc (%) (*n* = 50)	CS (%) (*n* = 90)	*p*
Age (years)^†^	48.7 (18-75)	48.3 (20-73)	0.9^∗^
Gender			
Female	88 (44)	94.4 (85)	0.2^∗∗^
Male	12 (6)	5.5 (5)	0.2^∗∗^
Disease subtype			
lcSSc	86 (43)	—	
dcSSc	14 (7)	—	
Disease duration (months)^†^	94.8 (3-420)	—	
Clinical manifestations			
Sclerodactyly	88 (44)	—	
Musculoskeletal damage	82 (41)	—	
Raynaud's phenomenon	70 (35)	—	
Puffy fingers	68 (34)	—	
Telangiectasia	54 (27)	—	
Digital ulcers	40 (20)		
Clinical evaluation			
HAQ^†^	0.4 (0-1.29)	—	
MRSS51^†^	7.3 (0-30)	—	

^†^Data are shown in median and rank in parentheses. SSc: Systemic Sclerosis; CS: Control Subjects; HAQ: Health Assessment Questionnaire disability index; MRSS51: Modified Rodnan Skin Score. ^∗^Mann–Whitney *U* test; ^∗∗^Fisher's test.

**Table 2:** *KIR* and *HLA* gene frequencies in SSc patients and CS.

	SSc (*n* = 50)			CS (*n* = 90)			*p* ^∗^	OR	95% CI	*p*′
	*n* CF	% CF	GF	*n* CF	% CF	GF				
*KIR2DL1*	47	94	0.76	87	96.7	0.82				
*KIR2DL2*	32	64	0.40	30	33.3	0.18	0.0007	3.6	1.7-7.3	0.011
*KIR2DL3*	46	92	0.72	87	96.7	0.82				
*KIR3DL1*	44	88	0.65	81	90	0.68				
*KIR3DL2*	50	100	1.00	88	97.8	0.85				
*KIR2DS1*	23	46	0.27	42	46.7	0.27				
*KIR2DS2*	18	36	0.20	28	31.1	0.17				
*KIR2DS3*	8	16	0.08	14	15.6	0.08				
*KIR2DS4*	41	82	0.58	80	88.9	0.67				
*2DS4full/del*	12	24	0.13	25	27.8	0.15				
*2DS4full*	18	36	0.20	51	56.7	0.34	0.02	0.4	0.2-0.9	0.09
*2DS4del*	12	24	0.13	4	4.4	0.02	0.001	6.8	2.1-22.4	0.021
*2DS4neg*	8	16	0.08	10	11.1	0.06				
*KIR2DS5*	20	40	0.23	37	41.1	0.23				
*KIR3DS1*	24	48	0.28	39	43.3	0.25				
*KIR2DP1*	46	92	0.72	87	96.7	0.82				
*KIR3DL3*	50	100	1.00	90	100	1.00				
*KIR2DL4*	50	100	1.00	90	100	1.00				
*KIR2DL5*	26	52	0.31	46	51	0.30				
*KIR3DP1*	49	98	0.86	90	100	1.00				
*HLA-C1*	41	82	0.58	82	91	0.70				
*HLA-C2*	37	74	0.49	48	53	0.31	0.02	2.5	1.2-5.3	0.09
*HLA-A^Bw4^*	48	96	0.80	75	83	0.59				
*HLA-Bw4^Iso80^*	11	22	0.12	23	26	0.14				
*HLA-Bw4^Thr80^*	5	10	0.05	7	8	0.04				
*HLA-A* ^∗^ *03*	4	8	0.04	10	11.1	0.06				
*HLA-A* ^∗^ *11*	3	6	0.03	1	1.1	0.005				

CF: Carrier Frequencies; GF: Gene Frequencies; *KIR*: Killer-cell Immunoglobulin-like Receptors; *HLA*: Human Leukocyte Antigen; SSc: Systemic Sclerosis; CS: Control Subjects; CI: Confidence Interval. *KIR2DS4full/del* includes individuals with a copy of the *full* gene and *del* variant; *2DS4full* includes individuals with one or two copies of the *full* gene; *2DS4del* includes individuals with one or two copies of the *del* variant; and *2DS4neg* includes individuals without *KIR2DS4*. *p*^∗^ based on Fisher's exact test and adjusted *p*′ based on Holm-Bonferroni correction for multiple comparisons.

**Table 3:** Combinations between the *KIR* gene and the *HLA-I* ligand.

*KIR/HLA*	SSc (%) (*n* = 50)	CS (%) (*n* = 90)	*p* ^∗^	OR	95% CI	*p*′
*KIR2DL2+/HLA-C1+*	54 (27)	32.2 (29)	0.02	2.5	1.2–5.0	0.08
*KIR2DL2+/HLA-C2+*	44 (44)	22.2 (20)	0.01	2.8	1.3–5.8	0.045
*KIR2DS4full+/HLA-C1+*	50 (25)	75.6 (68)	0.003	0.3	0.2–0.7	0.01
*KIR2DL2-/HLA-C1+*	28 (14)	58.9 (53)	0.001	0.3	0.1–0.6	0.004
*KIR2DL2-/HLA-C2-*	6 (3)	35.6 (32)	< 0.0001	0.1	0.03–0.4	0.0004
*KIR2DS4del-/HLA-C2-*	6 (3)	31.1 (28)	0.0005	0.1	0.04–0.5	0.002

*KIR*: Killer-cell Immunoglobulin-like Receptors; *HLA*: Human Leukocyte Antigen; SSc: Systemic Sclerosis; CS: Control Subjects. *p*^∗^ based on Fisher's exact test and adjusted *p*′ based on Holm-Bonferroni correction for multiple comparisons.

**Table 4:** *KIR* genotypes.

Genotype ID		*3DL1*	*2DL1*	*2DL3*	*2DS4*	*2DL2*	*2DL5*	*3DS1*	*2DS1*	*2DS2*	*2DS3*	*2DS5*	*2DL4*	*3DL2*	*3DL3*	*2DP1*	*3DP1*	SSc (%) (*n* = 50)	CS (%) (*n* = 90)
AA	1	—	—	—	—								—	—	—	—	—	14.0	37.8^∗^
AA	195	—	—	—									—	—	—	—	—	0.0	1.1
AA	203	—	—	—	—								—	—	—		—	2.0	0.0
Bx	2	—	—	—	—		—	—	—			—	—	—	—	—	—	10.0	18.9
Bx	3	—	—	—	—	—	—	—	—	—		—	—	—	—	—	—	4.0	5.6
Bx	4	—	—	—	—	—				—			—	—	—	—	—	8.0	5.6
Bx	5	—	—	—	—	—	—			—	—		—	—	—	—	—	4.0	3.3
Bx	6	—	—	—	—	—	—	—	—	—	—	—	—	—	—	—	—	0.0	1.1
Bx	7	—	—	—	—	—	—	—	—	—	—		—	—	—	—	—	0.0	2.2
Bx	8	—	—	—	—		—	—	—		—		—	—	—	—	—	2.0	3.3
Bx	9	—	—	—	—	—	—		—			—	—	—	—	—	—	2.0	3.3
Bx	18	—	—	—	—	—	—	—	—			—	—	—	—	—	—	8.0	1.1
Bx	19	—	—	—	—	—							—	—	—	—	—	14.0	0.0
Bx	31	—	—	—	—	—	—			—			—	—	—	—	—	2.0	1.1
Bx	58	—	—	—	—	—	—	—	—		—	—	—	—	—	—	—	2.0	0.0
Bx	68		—	—		—	—	—	—	—		—	—	—	—	—	—	0.0	1.1
Bx	69		—	—			—	—	—			—	—	—	—	—	—	2.0	2.2
Bx	70		—	—		—	—	—	—	—	—	—	—	—	—	—	—	2.0	0.0
Bx	71	—	—		—	—	—			—	—		—	—	—	—	—	2.0	1.1
Bx	72	—			**—**	—				—			—	—	—		—	0.0	1.1
Bx	74					—	—	—	—	—		—	—	—	—		—	2.0	1.1
Bx	75		—	—			—	—	—		—	—	—	—	—	—	—	0.0	2.2
Bx	76	—			—	—	—	—	—	—		—	—	—	—		—	2.0	0.0
Bx	88		—	—		—	—	—	—	—		—	—	—	—	—	—	6.0	1.1
Bx	117		—	—			—	—	—		—		—	—	—	—	—	0.0	1.1
Bx	275	—	—	—	—	—					—		—	—	—	—	—	2.0	0.0
Bx	331	—	—	—			—	—	—		—		—	—	—	—	—	2.0	0.0
Bx	336	—	—	—	—	—		—					—	—	—	—	—	0.0	1.1
Bx	384	—	—	—	—	—	—	—	—				—	—	—	—	—	2.0	0.0
Bx	475	—	—	—				—					—	—	—	—	—	2.0	0.0
Bx	476	—		—	—			—					—	—	—	—	—	2.0	0.0
Bx	620	—	—	—	—	—	—	—	—	—	—	—	—	—	—	—		0.0	1.1
Bx	651	—	—	—	**—**	—	—	—	—	—	—	—	—	—	—	—		0.0	1.1
Bx	653	—	—	—	—	—	—	—	—	—	—	—	—	—	—	—		0.0	1.1
Bx	No ID	—			—	—				—			—	—	—			2.0	0.0

Genotype ID was assigned according to the Allele Frequency Database [8]. Gene presence is indicated by a “—”, and its absence is indicated with empty spaces. The frequency of each genotype is presented in percentage. SSc: Systemic Sclerosis; CS: Control Subjects; AA: AA genotype; Bx: BB and AB genotypes. *∗* is based on Fisher's exact test (*p* = 0.03) and adjusted *p*′ based on Holm-Bonferroni correction for multiple comparisons (*p*′ = 0.02).

**Table 5:** Clinical manifestations and *KIR* genes.

	Sclerodactyly (%) (*n* = 44)	Musculoskeletal damage (%) (*n* = 41)	Raynaud's phenomenon (%) (*n* = 35)	Inflamed fingers (%) (*n* = 34)	Telangiectasias (%) (*n* = 27)	Digital ulcers (%) (*n* = 20)
*2DL2*	63.6 (28)	58.5 (24)	60.0 (21)	55.9 (19)	59.3 (16)	70.0 (14)
*2DS4full*	34.1 (15)	31.7 (13)	37.1 (13)	35.3 (12)	44.4 (12)	45.0 (9)
*2DS4del*	25.0 (11)	24.4 (10)	31.4 (11)	29.4 (10)	22.2 (6)	45.0 (4)
*2DS4full/del*	22.7 (10)	26.8 (11)	17.1 (6)	17.6 (6)	22.2 (6)	45.0 (4)
*2DS4neg*	18.2 (8)	17.1 (7)	14.3 (5)	17.6 (6)	11.1 (3)	15.0 (3)

*KIR2DS4full/del* includes individuals with a copy of the *full* gene and *del* variant; *2DS4full* includes individuals with one or two copies of the *full* gene; *2DS4del* includes individuals with one or two copies of the *del* variant; and *2DS4neg* includes individuals without *KIR2DS4*.

## Data Availability

The data used to support the findings of this study are available from the corresponding author upon request.
